# Carga laboral de profesionales de enfermería en Unidad de Cuidado Intensivo según Nursing Activities Score*

**DOI:** 10.15649/cuidarte.2680

**Published:** 2023-05-28

**Authors:** Ángela María Henao-Castaño, Juan David Melo-Roa, Jenny Fernanda Quintero-Osorio, Laura Nathalie Cruz-López

**Affiliations:** 1 . Universidad Nacional de Colombia. Bogotá, Colombia. Email: lncruzl@unal.edu.co Universidad Nacional de Colombia Universidad Nacional de Colombia Bogotá Colombia lncruzl@unal.edu.co; 2 . Universidad Nacional de Colombia. Bogotá, Colombia. Email: jfquinteroos@unal.edu.co Universidad Nacional de Colombia Universidad Nacional de Colombia Bogotá Colombia jfquinteroos@unal.edu.co; 3 . Universidad Nacional de Colombia. Bogotá, Colombia. Email: judmeloro@unal.edu.co Universidad Nacional de Colombia Universidad Nacional de Colombia Bogotá Colombia judmeloro@unal.edu.co; 4 . Universidad Nacional de Colombia. Bogotá, Colombia. Email: angmhenaocas@unal.edu.co Universidad Nacional de Colombia Universidad Nacional de Colombia Bogotá Colombia angmhenaocas@unal.edu.co

**Keywords:** Nursing Activities Score, Carga de Trabajo, Unidad de Cuidados Intensivos, Enfermería, Nursing Activities Score, Workload, Intensive Care Units, Nursing, Nursing Activities Score, Carga de Trabalho, Unidades de Terapia Intensiva, Enfermagem

## Abstract

**Introducción:**

Nursing Activities Score ha sido utilizada como un instrumento principalmente en la Unidad de Cuidados Intensivos para medir las actividades de enfermería, siendo esta la unidad que maneja pacientes de mayor complejidad para el cuidado.

**Objetivo:**

establecer la carga de trabajo, evaluada por Nursing Activities Score, y factores relacionados a la misma en Unidades de Cuidado Intensivo. Metodología: Revisión cualitativa tipo scoping Review, utilizando el método PRISMA. Búsqueda en las bases de datos CINAHL, LILACS, SCOPUS, SCIENCE DIRECT, SCIELO y PUBMED.

**Resultados:**

La muestra final se compone de 87 textos, que van desde el año 2007 hasta 2021. Se clasificaron en cinco categorías: Carga de trabajo en UCI, comparación entre unidades, carga de trabajo relacionada al personal de enfermería, carga de trabajo relacionada a las características de los pacientes y consecuencias de la carga de trabajo.

**Discusión:**

La revisión revela una carga de trabajo mayor al 50% en la mayoría de los estudios, esto debido a diferentes factores: principalmente las características particulares de los pacientes, no se observó una diferencia significativa entre unidades generales y especializadas, las cargas de trabajo elevadas suponen un factor de riesgo para la ocurrencia de eventos adversos.

**Conclusiones:**

Los resultados de esta revisión permiten evidenciar que el personal de enfermería está expuesto constantemente a altas cargas de trabajo. Esta carga de trabajo puede verse influenciada o influenciar diversos factores, como lo son las características de los pacientes a quienes se brinda atención o puede afectar positiva o negativamente la calidad de la atención de enfermería.

## Abstract (en)

**Introduction:**

The Nursing Activities Score has been used mainly in Intensive Care Units as an instrument to measure nursing activities since this is the unit that receives the most complex patients. For this reason, this scoping review aims to establish the workload using the Nursing Activities Score and the factors related to it in Intensive Care Units.

**Methods:**

Qualitative scoping review using the PRISMA statement. Searches were conducted in CINAHL, LILACS, Scopus, Science Direct, SciELO, and PubMed databases.

**Results:**

The final sample consists of 87 texts published between 2007 and 2021. They were classified into five categories: ICU workload, inter-unit comparison, workload related to nursing staff, workload related to patient characteristics, and workload consequences.

**Discussion:**

The review reveals a workload greater than 50% in most of the studies due to different factors, but mainly to patients' particular characteristics. No significant difference was observed between general and specialized units; high workloads are a risk factor for adverse events.

**Conclusions:**

This review shows that nurses are constantly exposed to high workloads. It is possible for workloads to affect or be influenced by various factors, such as the characteristics of patients being cared for, or to impact the quality of nursing care positively or negatively.

## Resumo

**Introdujo:**

O Nursing Activities Score tem sido usado como um instrumento principalmente na Unidade de Tratamentos Intensivos para medir as atividades de enfermagem, sendo essa a unidade que lida com os pacientes mais complexos para o cuidado. Por esta razao, o objetivo desta scoping review é estabelecer a carga de trabalho, conforme avaliado pelo Nursing Activities Score, e fatores relacionados em Unidades de Cuidados Intensivos.

**Metodologia:**

Revisao quantitativa usando scoping Review, utilizando o método PRISMA. Pesquisa nos bancos de dados CINAHL, LILACS, SCOPUS, SCIENCE DIRECT, SCIELO e PUBMED.

**Resultados:**

A amostra final consistiu em 87 textos, variando de 2007 a 2021. Eles foram classificados em cinco categorias: carga de trabalho na UTI, comparado entre unidades, carga de trabalho relacionada ao pessoal de enfermagem, carga de trabalho relacionada as características dos pacientes e consequéncias da carga de trabalho.

**Discussao:**

A revisao revela uma carga de trabalho superior a 50% na maioria dos estudos, isto devido a diferentes fatores: principalmente as características particulares dos pacientes, nenhuma diferenga significativa foi observada entre unidades gerais e especializadas, altas cargas de trabalho sao um fator de risco para a ocorréncia de eventos adversos.

**Condusdes:**

Os resultados desta revisao comprovam que o pessoal de enfermagem está constantemente exposto a altas cargas de trabalho. Esta carga de trabalho pode ser influenciada ou influenciada por vários fatores, tais como as características dos pacientes.

## Introducción

La herramienta Nursing Activities Score (NAS), creada por Miranda en el 2003^1^, es una escala diseñada para medir carga de trabajo en el personal de enfermería. Tiene 23 ítems, con puntuación entre 12 a 32 puntos porcentuales, permite calcular la carga de trabajo en tiempo de atención, cada punto corresponde a 14,4 minutos de asistencia. Esto permite tener información sobre los requerimientos de personal para el lugar donde ésta sea aplicada, incluye la evaluación de actividades del cuidado, como preparación y administración de medicamentos, actividades de monitoreo e higiene, igualmente contempla actividades de cuidado indirecto como la gestión de los medicamentos, labores gerenciales o administrativas.

Desde su implementación ha sido utilizada como un instrumento principalmente en la Unidad de Cuidados Intensivos (UCI) siendo esta la unidad que maneja pacientes de mayor complejidad y donde se hace necesaria la evaluación de la carga de trabajo y como se relaciona con resultados en salud.

La carga de trabajo es un concepto que se puede analizar desde diferentes aspectos como lo son físicos, psicológicos, ambientales y que van a modificar la forma en la que los individuos desempeñan una tarea en términos de productividad, calidad del producto o servicio y satisfacción del cliente.

El uso de la NAS en UCI se atañe principalmente a la medición de la carga de trabajo con las características del paciente^2^. Por este motivo, el objetivo de este scoping review es identificar la evidencia disponible sobre la carga de trabajo, evaluada por NAS, y factores relacionados a la misma en UCI.

## Materiales y Métodos

De acuerdo con el Instituto Joanna Briggs (JBI) un scoping review consiste en una revisión exploratoria, que permite identificar los tipos de evidencia disponible sobre determinado tema, así como los conceptos clave, límites y vacíos de investigación. El JBI propone las siguientes etapas para su desarrollo: Definir el título, establecer el trasfondo, revisar la pregunta y objetivo de investigación, establecer los criterios de inclusión bajo la estrategia Participantes, Concepto y Contexto (PCC), ejecutar la búsqueda, extraer y clasificar los resultados, discusión y conclusiones^3^.

Este estudio llevó un registro detallado de cada paso realizado de la metodología a través de una matriz elaborada en la plataforma Google Spreadsheets, teniendo en cuenta conceptos como: año de publicación, tamaño de la muestra, si la investigación hacía uso o no de NAS en algún tipo de UCI. Los criterios de inclusión fueron definidos con base en el objetivo del estudio (Tabla 1 y Tabla 2). La base de datos tiene acceso público en Mendeley Data^4^.

**Tabla 1:** Criterios de inclusión.

Criterio	Definición
Estrategia PCC	
Participantes	Textos en los que se haga uso de la herramienta Nursing Activities Score.
Concepto	Uso de la herramienta Nursing Activities Score y carga laboral de profesionales de enfermería
Contexto	Unidades de Cuidado Intensivo
	Criterio Definición
Otros criterios	
Tipo de texto	Textos científicos, artículos de investigación cuantitativos, cualitativos y mixtos, primarios y secundarios, de todos los niveles de evidencia disponible.
Idioma	Español, portugués, inglés e italiano.
Año	Sin límite de tiempo.

*Nota: Las búsquedas fueron ejecutadas por los investigadores entre el 1 de noviembre de 2021 y el 20 de noviembre de 2021.*

### Estrategia de búsqueda

Se realizó una búsqueda sin restricciones de tiempo en repositorios online identificando artículos relevantes que hayan usado de cualquier manera la escala NAS. (Tabla 3)

**Tabla 2:** Estrategias de búsqueda

Ítem	Aspectos vinculados
Palabras clave	Nursing Activities Score, NAS, workload, nursing staff, hospital, carga de trabajo.
Repositorios	CINAHL, LILACS, SCOPUS, SCIENCE DIRECT, SCIELO y PUBMED.
Registro de búsqueda	Se utilizó la herramienta (PRISMA-ScR) completando las búsquedas en los repositorios
Ecuaciones	"nursing activities score AND unidad de terapia intensiva AND scoping review" “nursing activities score AND ICU” “enfermagem AND carga de trabalho AND nas OR Nursing Activity Score”

*Nota: Utilizando combinaciones de términos clave. Fuente: Page MJ, McKenzie JE, Bossuyt PM, Boutron I, Hoffmann TC, Mulrow CD, et al. The PRISMA 2020 statement: An updated guideline for reporting systematic reviews. BMJ. 2021;372.*

**Figura 1:**
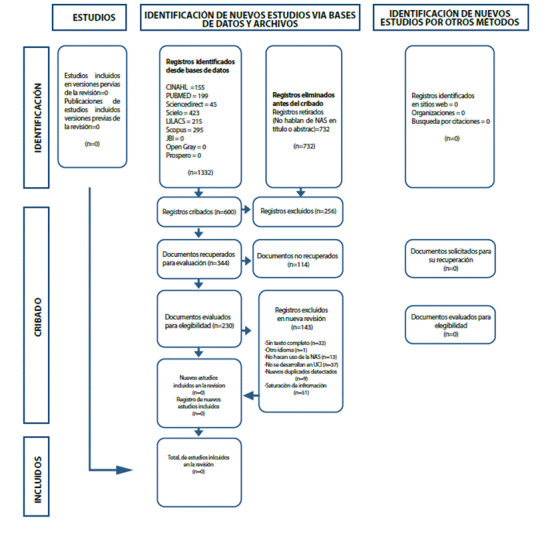
Diagrama de flujo PRISMA.

## Resultados

Partiendo de la estrategia de búsqueda, se obtuvieron 1332 resultados. Únicamente 87 de los 230 textos seleccionados para realizar lectura cumplen con los criterios de elegibilidad establecidos en esta revisión, constituyendo la muestra final. Estos fueron caracterizados y clasificados utilizando la matriz descrita anteriormente.

### Características de los recursos revisados

Los recursos encontrados fueron artículos publicados en revistas de diferentes nacionalidades y años. (Tabla 3)

**Tabla 3:** Clasificación de los recursos encontrados

Aspecto	Hallazgos
País de origen	Brasil el país con mayor producción de textos (78.2%). Entre otras se encuentran España, Grecia, Bélgica, Holanda, Noruega, Colombia, Italia, Irán, Polonia.
Diseño del texto	Se halló una distribución 96.6% de tipo cuantitativo y 3.4% de tipo cualitativo.
Año	Los textos van desde el año 2007 hasta el año 2021
Proporción tipo de UCI donde se aplicó la NAS	UCI general con 67.8%, neonatal 8%, cardiología 6.9%, varios (manejan más de un tipo de UCI) 4.6%, pediatría 3.4%, trauma 3.4%, unidad de cuidados intermedios 2.3%, neurología 1.1%, quemados 1.1% y oncológica 1.1%.

### Clasificación de recursos revisados

Se realizó clasificación de los textos según el desempeño que tenía la carga de trabajo dentro del contenido, resultando cinco categorías.

1. *Carga de trabajo en UCI*
2. *Comparación entre unidades*
3. *Carga de trabajo relacionada al personal de enfermería*
4. *Carga de trabajo relacionada a las características de los pacientes*
5. *Consecuencias en la carga de trabajo*

### Carga de trabajo en UCI

Maziero et al., menciona que “NAS es un instrumento valioso para establecer un equilibrio entre la carga de trabajo y los profesionales disponibles, siendo más preciso que las disposiciones de la legislación brasileña sobre Dimensionamiento del Personal de Enfermería”^18^.

A través del uso de la NAS se encontraron valores mayores al 52% de puntuación media encontrando el valor más bajo en 52,5% (menor carga laboral encontrada) y el más alto 93,1% (mayor carga laboral encontrada)^5^^,^^6^^,^^7^^,^^8^^,^^11^^,^^13^^,^^14^^,^^15^^,^^18^^,^^21^^, ^^25^^, ^^26^_._

Sin embargo, Inoue et al. en su estudio, con resultados similares en la aplicación de la NAS a los otros estudios, refieren que la carga de trabajo de enfermería se acerca al valor referente a la “alta carga”, y aún es necesario repensar la dinámica de ingreso de los pacientes a este servicio, ya que, entre las variables investigadas, el tratamiento quirúrgico es la única que interfiere directamente en el aumento de la carga de trabajo^10^.

### Comparación entre unidades

En los recursos revisados se encuentran seis ^27^^,^^28^^,^^29^^,^^30^^,^^31^^,^^32^ que realizan comparación de la carga de trabajo entre diferentes tipos de unidades. Bochembuzio^28^ buscó identificar las diferencias en la carga de trabajo en una unidad neonatal (UN) y una unidad de terapia intensiva neonatal (UTIN), obteniendo como resultado que la UTIN tiene una mayor carga de trabajo que la UN (90% y 67% de tiempo de cuidado respectivamente) e identificó la NAS como un instrumento adecuado para medir la carga de trabajo del equipo de enfermería en el área neonatal.

Queijo^28^ analizó la carga de trabajo entre UCI general, cardiológica y neurológica, para las cuales obtuvo un puntaje de 66.54%, 66.66% y 65.18% respectivamente, mostrando una diferencia no significativa. Un trabajo similar fue realizado por Luccini et al.^29^, donde los resultados medios de la NAS de la UCI general, neurológica y cardiotorácica fueron de 72.55%, 59,33% y 63,51% respectivamente, mostrando diferencias significativas. Por otro lado, Santos Nobre et al.^30^ comparó, en una revisión sistemática de 20 publicaciones, la carga de trabajo entre UCI general, quirúrgica, cardiológica y de trauma, obteniendo como resultado que en 17 ocasiones la carga de trabajo superó el 50%.

El estudio de Nogueira et al.^31^ comparó la carga de trabajo de cuatro UCI públicas con dos de tipo privado, en el cual hubo diferencia estadísticamente significativa (68.1 puntos en públicas y 56 puntos en privadas) en la carga de trabajo de enfermería entre los pacientes de las unidades públicas y privadas para las primeras 24 horas de estancia, con los puntajes NAS más altos exhibidos por los pacientes de las unidades públicas.

Cyrino et al.^32^ comparó la carga de trabajo del sitio postoperatorio, sitio de aislamiento y el sitio de estancia prolongada de un UCI, la comparación de distintos meses demostró que “las actividades del cuidado de enfermería pueden cambiar, dependiendo del grado de dependencia del paciente, de la complejidad de la enfermedad, de las características de la institución, organización y procesos de trabajo del equipo”.

### Carga de trabajo relacionada al personal de enfermería

De acuerdo con las mediciones realizadas con la NAS, el personal administrativo puede calcular los profesionales requeridos para cada unidad mejorando la gestión del recurso humano^33^^,^^34^^,^^35^^,^^38^^, ^^39^^,^^40^^,^^41^^,^^42^^,^^52^. De igual forma el número de profesionales disponibles por paciente garantiza la calidad de la atención. Los costos en la atención no se manejan únicamente por número de profesionales, el número de horas de cuidado directo e indirecto^37^ es una medida que también permite el cálculo del equipo de enfermería. Las cargas mentales de los profesionales de enfermería son altas al igual que la carga física evaluada por la NAS^43^.

### Carga de trabajo relacionada a las características de los pacientes

En esta revisión se relacionan diversas características de los pacientes con la carga de trabajo. Una de las características principales es la edad donde la carga de trabajo aumenta en pacientes mayores de 70 años^44^^, ^^45^^, ^^58^^, ^^65^ la variable “sexo” también aumento la carga de trabajo en el caso de los hombres^67^, el nivel de complejidad, o condiciones de salud específicas que los lleven a requerir de una atención especializada y continua^46^^, ^^50^^, ^^52^^, ^^54^^, ^^56^^, ^^57^^, ^^58^^, ^^59^^, ^^63^^, ^^68^^, ^^69^^, ^^70^^, ^^73^^, ^^74^^, ^^75^^, ^^76^^, ^^77^^, ^^78^_,_ supone un aumento en las cargas de trabajo.

El pronóstico con índices de mortalidad y disfunción orgánica guardan correlaciones positivas con el aumento de la carga de trabajo^47^^,^^53^^,^^55^, pues, dependiendo de dichas variables, el paciente requiere de cuidados que se reflejan en la puntuación de los ítems de la escala.

Otros estudios encuentran una relación significativa en la carga de trabajo al momento de egreso, pues los pacientes que fallecen puntúan mayor NAS en comparación con los sobrevivientes^48^^,^^49^^,^^67^. Adicionalmente se observa una relación significativa entre las estancias hospitalarias prolongadas^73^ y una puntuación NAS mayor. Si bien la puntuación NAS será mayor al ingreso, tiende a disminuir con el tiempo de estancia^51^, adicionalmente si la estancia hospitalaria se prolonga puede que aumente el puntaje NAS. En contraste también se encuentran estudios que contradicen las premisas anteriores^59^^, ^^60^^,^^61^^,^^64^^,^^66^^,^^71^ pues identifican relaciones moderadas o bajas entre la carga de trabajo y las variables de edad, estancia, gravedad y riesgo de mortalidad.

### Consecuencias de la carga de trabajo

De Oliveira et al. concluye que la alta carga de trabajo requerida por los pacientes en UCI se identificó como un factor de riesgo para la aparición de úlceras por presión y/o errores de medicación en seis de los ocho estudios examinados^86^.

Sobrinho observó que los pacientes graves exigen una mayor carga asistencial. Así, la relación entre el NAS y el SAPS (mide la gravedad de los pacientes) es significativa al analizar las variables puntuadas en cada una de las herramientas. Así mismo, aquellos pacientes con mayores complicaciones son los que mayor número de intervenciones requieren. Sin embargo, el aumento de la carga de trabajo también aumenta el riesgo de fallos durante la asistencia^90^^).^

Como hallazgo general los artículos encontrados concuerdan en que los pacientes que permanecen más tiempo en UCI con alta gravedad clínica y una condición inestable, también requirieron mayor seguimiento, soporte ventilatorio, cardiovascular, y más intervenciones específicas y, por tanto, más tiempo y actividades de enfermería medidas por la NAS.^80^^, ^^81^^, ^^82^^, ^^83^^, ^^84^^, ^^85^^, ^^86^^, ^^87^^, ^^88^^, ^^89^^, ^^90^^, ^^91^

## Discusión

Los resultados en esta revisión reafirman que la carga de trabajo en las UCI es mayor al 50%. Lo que indica que un profesional solo brinda cuidado de manera integral a un paciente por turno de trabajo. Por esto, la medición de la carga de trabajo tiene gran relevancia para calcular el número de profesionales y tiempo que se invierte realizando acciones de cuidado, convirtiendo a la NAS en una herramienta para la gestión del recurso humano.

Aquellos estudios que comparan distintos tipos de unidades no demuestran una gran diferencia entre las unidades generales y especializadas.

La puntuación NAS es modificada según varían las actividades, dependiendo del grado de dependencia del paciente, complejidad y demás variables exploradas en esta revisión. Las características de los pacientes son variables que afectan la carga de trabajo. En esta revisión se observa que los estudios buscan identificar cuáles características son más relevantes, las más estudiadas son la complejidad o gravedad del paciente que se apoyan de instrumentos que permiten evaluar el estado del paciente. Las características de la institución y de la organización y procesos de trabajo del equipo^31^ también son factores que modifican la carga de trabajo. En relación con los profesionales, algunos de los factores que pueden afectar la carga de trabajo son el tipo de turnos de trabajo, sexo del profesional, tipo de UCI, el número de pacientes a cargo, entre otros.

La mayor parte de textos que dan uso a la NAS tiene lugar en Brasil. Estos documentos valoran la carga de trabajo, y comparan sus resultados con otros textos, basando su clasificación de la carga de trabajo en diferentes variables como: clasificación del paciente, origen de la admisión y tipos de unidades hospitalarias^92^. En general el uso de diferentes instrumentos de medición de carga de trabajo en la UCI es de gran relevancia, así como la investigación acerca del uso de estos instrumentos, los estudios como las revisiones integrativas permiten dilucidar el uso adecuado de estas herramientas mediante su comparación^22^^,^^93^.

Si bien es importante identificar las ventajas de hallar literatura, documentos, estudios o investigaciones, en donde se trabaje o se exploren nuevos usos de la herramienta NAS, en la búsqueda expuesta en este documento no se exploró por completo todo el material disponible en los reservorios online puesto que se descartaron documentos en relación a su idioma y el lapso de tiempo en el que se realizaron las búsqueda (entre el 1 de noviembre de 2021 y el 20 de noviembre de 2021), lo anterior supone unas limitaciones en relación a las conclusiones puesto que los documentos encontrados y clasificados provienen, en su mayoría, de contextos muy similares por lo que hubiera sido más nutritivo para el documento analizar y clasificar documentos provenientes de diferentes países con contextos sociales y laborales completamente diferente a los latinoamericanos o suramericanos (de donde provienen la mayoría de textos encontrados para esta búsqueda) que hubieran podido complementar y contrastar resultados de la herramienta NAS identificando si las diferencias sociales son un determinante importante para los resultados de la carga laboral usando NAS como herramienta de medición y así mismo reconocer si el uso de la NAS también resulta útil fuera de la UCI.

Sin embargo, consideramos importante este documento y lo que se encontró aquí para acercarse al uso actual de la NAS en contextos sociales muy similares, como el sudamericano, para identificar puntos fuertes, debilidades, y, más importante aún, la carga de trabajo en servicios (UCI, HOSPITALIZACION, UCI ESPECIALIZADAS) para entender la situación y el trabajo actual del personal de enfermería y a su vez contrastar estos resultados con las labores esperadas o ideales para enfermería.

## Conclusiones

Los resultados de esta revisión evidencian que el personal de enfermería está expuesto a altas cargas de trabajo que, en la mayoría de los casos, supera lo sugerido por sus consejos y asociaciones. La carga de trabajo puede verse influenciada por diversos factores, como las características de los pacientes, situaciones propias de cada institución o país, así como las prácticas de enfermería realizadas. De igual forma, la carga de trabajo puede afectar la calidad de la atención, llegando a tener efectos en la presencia de eventos adversos, morbilidad y mortalidad de los pacientes que recibieron atención del personal.

Es importante reconocer que para identificar el trabajo actual de enfermería y sus actividades es necesaria la comparación de uso y resultados de NAS en contextos similares (como los presentados en este documento) y, así mismo, se sugiere contribuir con más material, en la comparación de esos resultados con documentos de tengan el uso de la NAS en otro tipo culturas con características diferentes (como Asia o países árabes)

Resumiendo, las contribuciones y conclusiones de todos los artículos integrados en esta revisión, es evidente que la herramienta NAS es relevante y brinda información del trabajo de enfermería en diferentes entornos.
